# Molecular Dynamics Study on Solution Transport Properties in CSH/SiO_2_ Nanochannels Under High-Temperature and Water-Rich Environments

**DOI:** 10.3390/ma19173766

**Published:** 2026-09-04

**Authors:** Yong Chen, Yiwei Hu, Xianjie Weng, Zhou Lv, Xing Liu, Xiaochen Wang, Lianzhen Zhang

**Affiliations:** 1 Jiangxi Communications Investment Group Co., Ltd., Nanchang 330108, China; 2 College of Pipeline and Civil Engineering, China University of Petroleum, Qingdao 266580, China; 3 School of Civil Engineering, Shandong Jianzhu University, Ji’nan 250101, China

**Keywords:** CSH/SiO_2_ nanochannels, high-temperature environment, water-rich environment, molecular dynamics simulation, transport behavior

## Abstract

This study employs molecular dynamics simulations to construct a CSH/SiO_2_ nanochannel model, systematically investigating the dynamic transport behavior of pure water and Na_2_SO_4_ solution within the pores across temperatures ranging from 293 K to 368 K. The results elucidate the fluid intrusion mechanism under the combined effects of elevated temperature and sulfate ions. Results show that: (i) Water migration within the CSH/SiO_2_ channel exhibits wall-dependent differences. The interaction between water and the CSH surface is approximately eight times stronger than with the SiO_2_ side at 293 K, causing water molecules to preferentially advance along the CSH side. As the penetration depth difference between the two sides increases, local water molecules extend toward the SiO_2_ side, forming liquid finger-like protrusions that lead to rapid filling on the SiO_2_ side. (ii) Elevated temperature enhances water molecule mobility; at 368 K, the mean square displacement of water in the pure water system is about 3.7 times greater than at 293 K, and the critical time for liquid finger formation on the SiO_2_ side decreases from 700 ps at 293 K to 100 ps at 368 K. (iii) The introduction of Na_2_SO_4_ solution alters the transport mechanism within the nanochannel. Na^+^ and SO_4_^2−^ form ionic coordination structures with oxygen and calcium sites on the CSH surface, establishing Na-O_CSH_ and S-Ca_CSH_ ionic bonds, which reduces the solution’s transport rate. Na^+^ and SO_4_^2−^ adsorb and accumulate near the CSH/SiO_2_ interface; heating weakens their hydration shells and strengthens the ion association between Na^+^ and SO_4_^2−^, promoting the formation of local ion clusters.

## 1. Introduction

As underground space development continues to advance into deeper regions, high temperatures and water-rich fractured environments have gradually become critical factors affecting the safe operation of tunnel projects [1,2,3]. Grouting reinforcement is a primary technical method for enhancing the integrity of fractured rock masses and reducing permeability and has been widely applied in deep underground engineering. Due to their high strength, durability, and cost-effectiveness, cement-based materials have become the preferred choice for grouting applications [4,5,6]. The grout–rock interface represents the weakest zone within the grouting reinforcement system, typically characterized by high porosity and weak structural bonding, making it the primary pathway for water solutions and corrosive ions to penetrate into the grouted body [7,8]. Under deep, high-temperature, and water-rich conditions, continuous intrusion of aqueous solutions and sulfate ions further exacerbates interface degradation, thereby compromising the long-term stability of the grouting system [9,10,11]. Therefore, understanding the migration mechanisms of water and ions at the grout–rock interface under high-temperature and sulfate-rich groundwater conditions is essential for improving the durability of deep grouting projects.

Numerous experimental studies have been conducted on the degradation behavior of grout–rock interface under high-temperature and groundwater erosion conditions. Sun [12] investigated the influence of the interfacial transition zone (ITZ) on the permeability of grout–rock composite systems through permeability tests. Due to its higher porosity, the ITZ exhibits significantly greater permeability than the dense matrix, making it a primary pathway for moisture migration. Wang [13] further studied the evolution of the ITZ under groundwater exposure via seepage and dissolution experiments at rock-concrete interfaces, revealing that C-S-H and other calcium-containing hydration products gradually dissolve, increasing interfacial porosity and forming dominant flow paths. Barbara [14] found through cement hydration experiments under different temperatures that elevated temperatures accelerate early hydration but also lead to more uneven distribution of hydration products and coarsening of pore structures. Tong [15] analyzed the effects of high temperature on grouting interfaces using SEM testing, showing that as curing temperature increases, the connectivity of cement hydration products deteriorates, micro-pores and micro-cracks progressively develop, thereby reducing the shear strength of grouted joints and interface integrity. Liu et al. [16] conducted sulfate attack experiments demonstrating that continuous intrusion of SO_4_^2−^ induces decalcification and structural disintegration of C-S-H gel, with high-temperature environments significantly accelerating both the rate of sulfate attack and penetration depth. These studies indicate that both high temperature and groundwater erosion degrade the structure of the grout–rock interface and alter its water and ion transport properties.

Traditional experiments have primarily focused on the macroscopic property changes in cement-based materials after fluid intrusion, such as reduced compressive strength and increased permeability [17,18,19]. Limited by observational techniques, these studies struggle to capture the dynamic transport of water molecules or the interfacial adsorption distribution of ions at the nanoscale. Molecular dynamics (MD) simulations, capable of tracking particle motion at the atomic level, have become an effective tool for investigating ion adsorption, diffusion, and interfacial interactions within C-S-H gel pores [20,21]. Results show that water molecules in nanopores differ significantly from bulk water in terms of migration rate, ion hydration structure, and hydrogen bonding networks-mainly due to electrostatic interactions and geometric confinement effects at the C-S-H surface [22,23,24,25]. Hou et al. [25] used MD simulations to study the adsorption and diffusion behaviors of SO_4_^2−^ and Cl- in C-S-H gel, finding that aggressive ions can interact with active sites on the C-S-H surface, thereby affecting the structural stability of the gel. Luan et al. [26] discovered that Na^+^-SO_4_^2−^ ion clusters can block the nanochannels of C-S-H gel, significantly slowing down water molecule transport. Wang et al. [27] systematically investigated the transport behavior of Na_2_SO_4_ solutions through C-S-H nanopores across a temperature range of 300–390 K. Heating accelerated capillary-driven water movement but also promoted the formation of ion clusters at the pore walls. These studies provide important foundations for understanding fluid transport in nanopores. However, systematic atomic-scale investigations into the dynamic capillary transport mechanisms of fluids in CSH/SiO_2_ nanocracks under combined high-temperature and sulfate ion conditions remain lacking.

This study systematically investigates the effects of temperature and solution type on fluid transport behavior within the CSH/SiO_2_ nanochannels using molecular dynamics simulations. By constructing a CSH/SiO_2_ nanochannel model, dynamic transport processes of pure water and Na_2_SO_4_ solution were simulated, and quantitative analyses of transport behaviors at six different temperatures were conducted to explore the mechanisms by which temperature and sulfate ions influence fluid transport. Based on these results, the study focuses on analyzing asymmetric wetting characteristics, cavity filling processes, as well as the spatial distribution and adsorption behavior of Na^+^ and SO_4_^2−^ ions within the channels. From a micro-scale transport perspective, this work reveals the infiltration mechanism of groundwater into nanoscale fractures at the grout–rock interface, providing theoretical support for designing impermeable and durable grouting structures in deep, high-temperature, water-rich environments.

## 2. Model Construction and Simulation Methods

### 2.1. Model Construction

**Figure 1:**
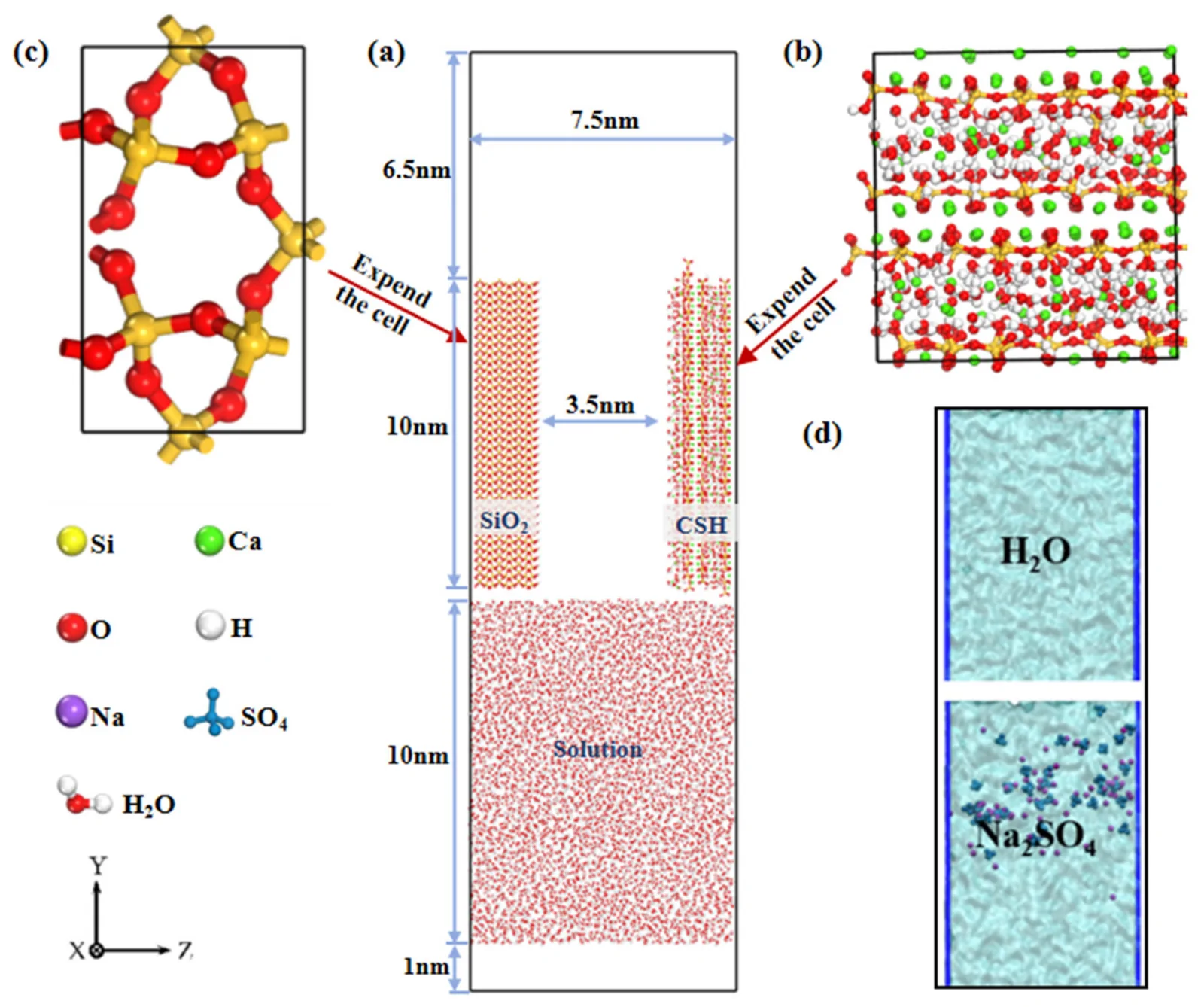
Models construction process (**a**) CSH/SiO_2_ nanochannel model, (**b**) CSH matrix, (**c**) SiO_2_, (**d**) solution cases: H_2_O, Na_2_SO_4_ solution.

The CSH/SiO_2_ nanochannel model is shown in Figure 1, consisting primarily of a CSH/SiO_2_ nanochannel and two aqueous solutions. Calcium silicate hydrate (CSH) accounts for approximately 60% of the volume of cement hydration products, and CSH gel structures are commonly used to represent cement paste in studies [28,29]. Tobermorite shares a layered calcium silicate framework similar to that of CSH gel; by adjusting the degree of polymerization of silicon chains and the Ca/Si ratio, it can effectively simulate the structural characteristics of actual cement hydration products [30]. In this study, following the methods proposed by Pellenq et al. [31] and Hou et al. [32], an 11 Å Tobermorite crystal structure was adopted as the basis. By randomly removing bridging SiO4 tetrahedra and adding ions, the Ca/Si ratio was adjusted to around 1.7. The final CSH gel supercell formula was determined as (CaO)_1.69_(SiO_2_)(H_2_O)_1.8_. On the rock substrate side, SiO_2_ crystals were selected to represent the major mineral components in sandstone and granite [33]. By expanding the SiO_2_ unit cell and cutting along the [0 0 1] direction to expose active surfaces, hydrogenation was applied to unsaturated oxygen atoms on the surface to create a hydrated surface containing silanol groups [34,35], as illustrated in Figure 1c.

After the pre-treatment of each component, the CSH surface was placed parallel to the SiO_2_ surface, forming a nanopore with a width of 3.5 nm. Two different aqueous solutions were selected in this study: pure water and a 5% mass fraction Na_2_SO_4_ solution, as shown in Figure 1d. Sulfate is one of the most representative aggressive ions in groundwater and significantly damages the durability of cement-based materials; therefore, Na_2_SO_4_ solution was chosen as a representative medium to simulate groundwater corrosion. The Na_2_SO_4_ solution model contains 4944 water molecules, 72 sodium ions, and 36 sulfate ions. Water molecules were modeled using the SPC model, which offers both high accuracy and computational efficiency in simulations of cement-based materials and electrolyte solutions [36]. To clarify the fundamental microscopic mechanisms of fluid transport within the nanochannel, the model neglected macroscopic roughness and heterogeneity at the interface [37]. A vacuum space was set on the top and bottom of the box to avoid water molecules escaping along y direction [38].

### 2.2. Force Field Selection and Simulation Workflow Details

All atomic-scale models were built using Visual Molecular Dynamics (VMD) [39], while molecular dynamics simulations were carried out with the LAMMPS package (version dated 8 February 2023) [40]. Trajectory analysis and real-time visualization of dynamic transport processes were conducted within the VMD environment.

**Figure 2:**
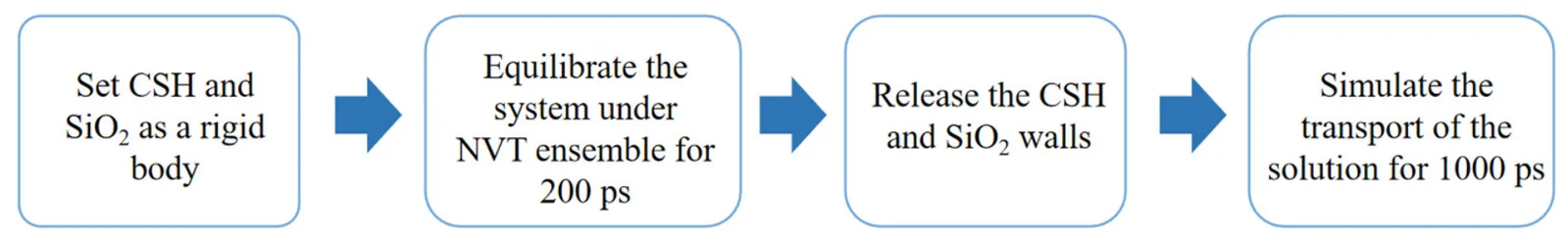
Flowchart of the simulation process.

This study employs the ClayFF force field as the computational model [41,42], which has been extensively validated for simulating silicate mineral structures, interfacial hydration, and solution transport behavior. The cutoff radius for van der Waals and short-range electrostatic interactions is set to 10 Å, while long-range electrostatic contributions are accurately calculated using the PPPM method [43]. Temperature is controlled by a Nose-Hoover thermostat, with an integration time step of 1 fs. All simulations are performed under periodic boundary conditions to eliminate finite-size effects. Each interface model undergoes identical simulation procedures at six target temperatures: 293 K, 308 K, 323 K, 338 K, 353 K, and 368 K. Initially, the CSH and SiO_2_ walls are fixed as rigid bodies to prevent channel deformation from interfering with fluid transport [44]. Subsequently, an invisible wall is placed between the channel and the solution to prevent water molecules and ions from entering the channel prematurely. The bottom solution is relaxed for 200 ps under the NVT ensemble to achieve thermodynamic equilibrium. Before fluid intrusion, the rigid constraints on the CSH and SiO_2_ surfaces were removed, and the simulation was performed under the NVT ensemble to achieve the equilibrium state of the substrates. Finally, the entrance barrier is removed, allowing the solution to enter the CSH/SiO_2_ nanochannel, a process simulated for 1000 ps under the NVT ensemble, as shown in Figure 2. During the simulation, atomic coordinates and energy data are recorded every 0.1 ps for subsequent quantitative analysis of fluid flow, density distribution, and radial distribution functions.

## 3. Results and Discussion

### 3.1. Transport of Water and Ions in CSH/SiO_2_ Channels

**Figure 3:**
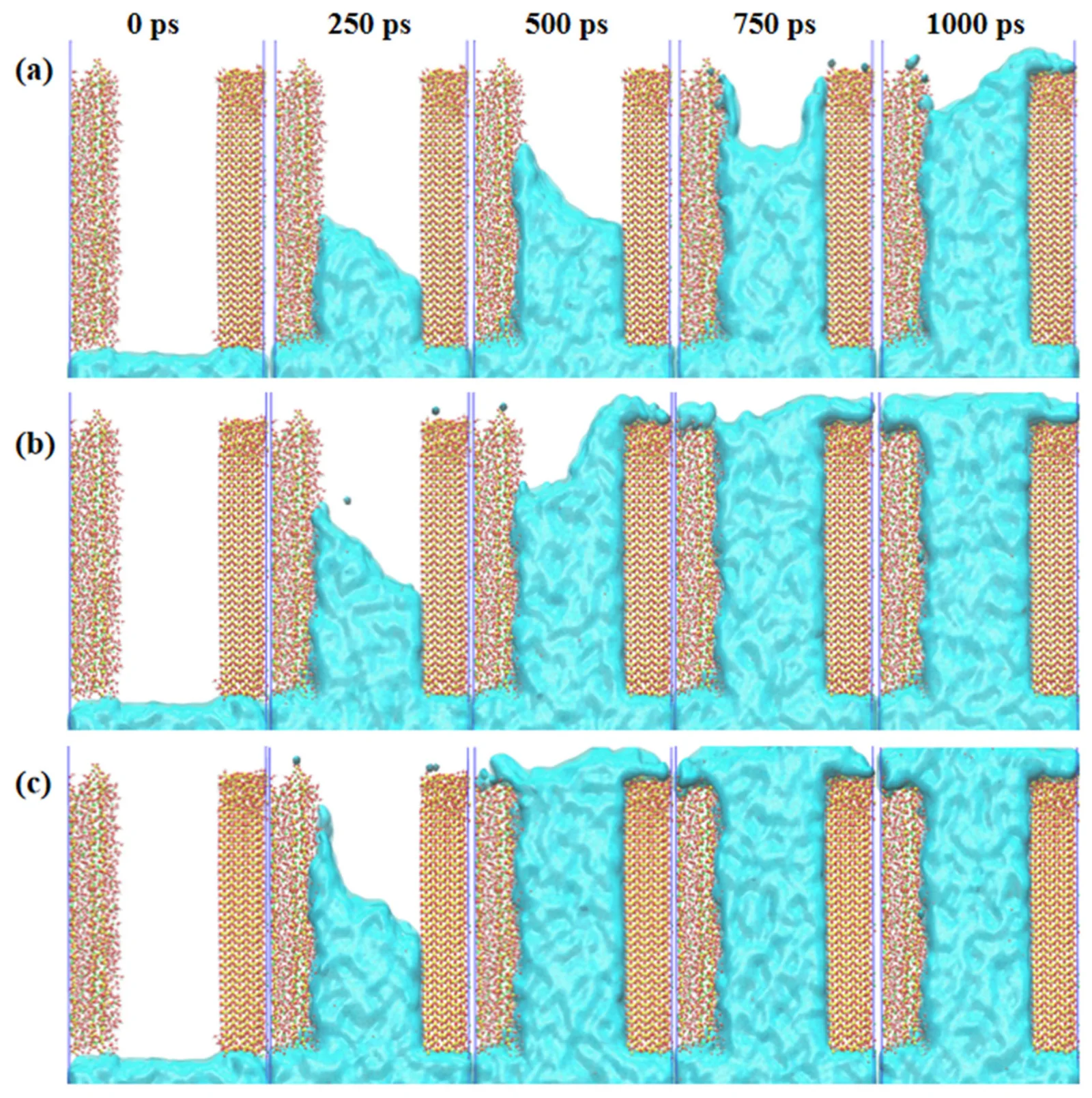
Snapshots of capillary flow of water (**a**) 293 K; (**b**) 323 K; (**c**) 353 K.

Figure 3 shows molecular snapshots of pure water solutions at different temperatures. In the pure water environment, water molecules diffuse and migrate into the CSH/SiO_2_ nanochannels starting from the bottom of the channels. As migration time increases, the liquid front continuously advances inward, forming a distinct liquid intrusion region. During the initial stage (0–500 ps), differences in water molecule migration behavior are observed on the two channel walls: the climbing height of water molecules on the CSH side is higher than that on the SiO_2_ side across all three temperatures, resulting in an overall asymmetric liquid surface tilted from the CSH side toward the SiO_2_ side, rather than the symmetric concave meniscus typical of classical capillary transport. With increasing temperature, water molecule migration on both substrate sides becomes enhanced, indicating that elevated temperature accelerates the transport rate of the aqueous solution.

**Figure 4:**
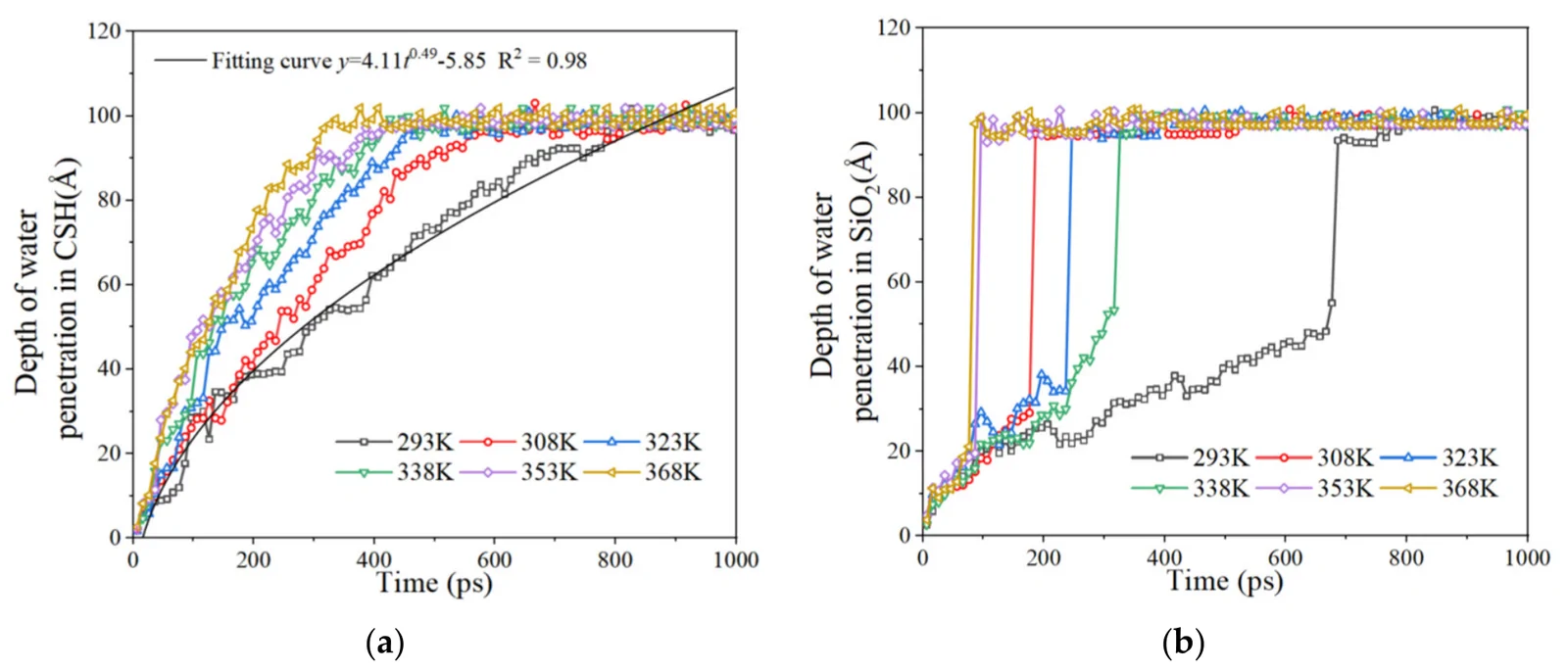
Penetration depths of water along the (**a**) CSH surface and (**b**) the SiO_2_ surface.

The maximum migration distance of water molecules on the CSH and SiO_2_ sides represents the fluid penetration depth, and the penetration depths in both matrix sides are shown in Figure 4. In the early stages of simulation, the penetration depth on the CSH side increases significantly faster than that on the SiO_2_ side, consistent with the observation from snapshots showing preferential migration of water toward the CSH side. The penetration depth on the CSH side exhibits a parabolic growth trend over time, matching the behavior predicted by the classical Lucas-Washburn capillary transport equation [45]. To quantitatively verify this, the penetration depth on the CSH side at 293 K was fitted using the Lucas-Washburn equation, yielding n = 0.49 and R^2^ = 0.98. This result is in excellent agreement with the theoretical exponent value of 0.5. This confirms the accuracy of using the ClayFF force field for predicting capillary absorption in MD simulations.

The migration behavior of the aqueous solution on the SiO_2_ side exhibited distinct characteristics, as shown in Figure 4b. At 293 K, the penetration depth increased slowly at first, following a gradual parabolic trend up to 700 ps, after which it rose rapidly, reaching 95 Å within 10 ps and remaining stable thereafter. Under higher temperatures, this abnormal rapid migration also occurred, with the water migration rate increasing as temperature rose. At 308 K and 323 K, the penetration depth reached a stable state after approximately 300 ps and 200 ps, respectively. When the temperature was further elevated to 338 K, 353 K, and 368 K, water molecules quickly migrated to the end of the SiO_2_ region within about 100 ps, stabilizing the penetration depth at 100 Å.

**Figure 5:**
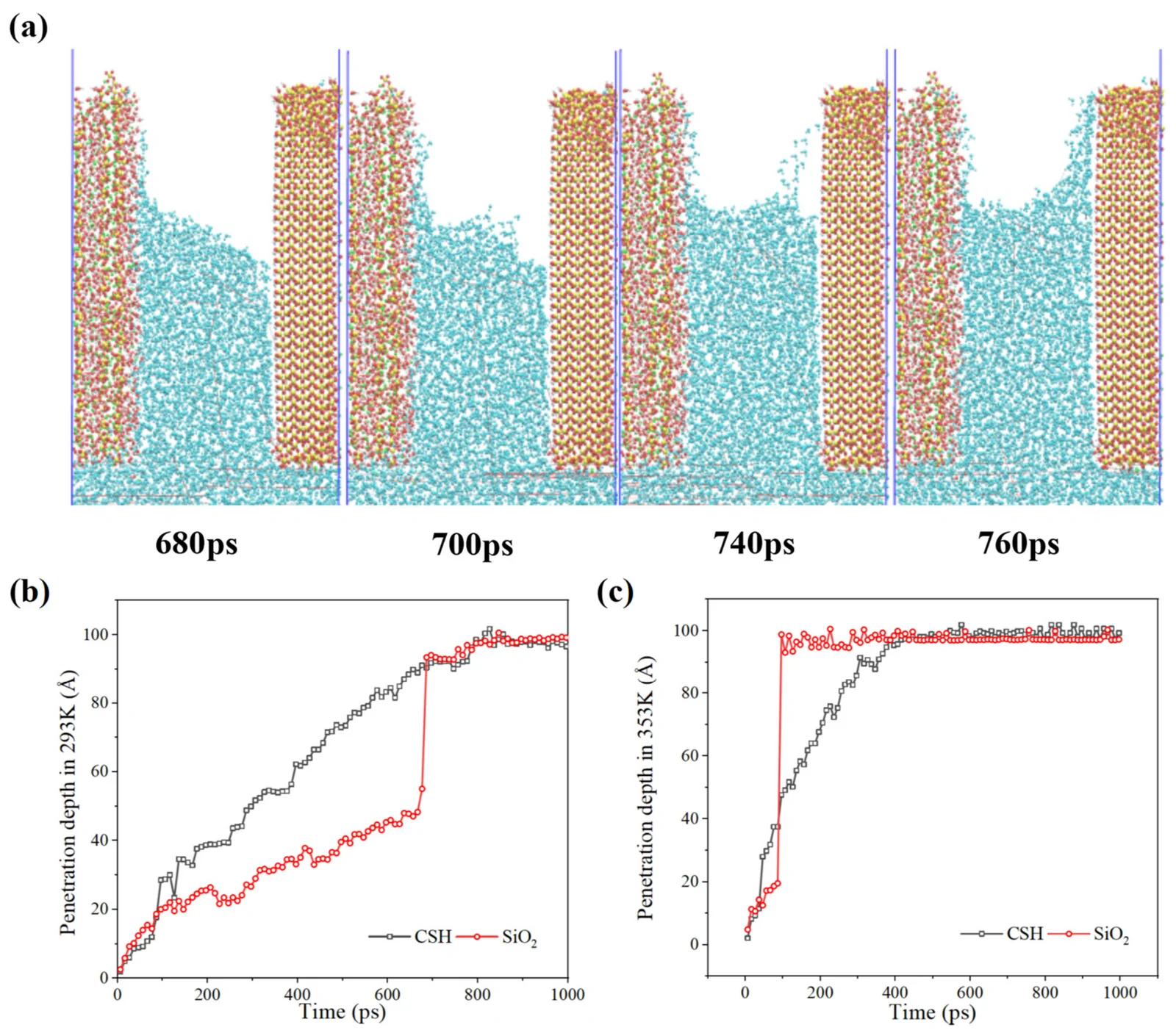
(**a**) Snapshots of the asymmetrical wetting process and variations in water penetration depth along CSH and SiO_2_ substrates at (**b**) 293 K and (**c**) 353 K.

To investigate the rapid migration phenomenon of aqueous solution near the SiO_2_ surface, molecular snapshots were analyzed during the time interval when water molecules showed a sudden increase in rising height. Figure 5 shows snapshots of aqueous solution migration at the SiO_2_ side within the 680–760 ps time window at 293 K, along with comparisons of penetration depth at different temperatures. At 680 ps, the adsorption state exhibited higher water concentration on the CSH side and lower on the SiO_2_ side. By 700 ps, a portion of water molecules protruded from the center of the channel toward the SiO_2_ side; around 740 ps, these water molecules formed a liquid-finger-like structure connected to the elevated region on the SiO_2_ side, creating a cavity. Subsequently, a large number of water molecules rushed into this cavity, forming a continuous migration channel by 760 ps, leading to rapid expansion of the infiltration zone along the SiO_2_ surface.

To verify the statistical reliability of the critical time for liquid finger-like structures, three independent simulation runs were performed at 293 K and 368 K using different initial velocity seeds. The repeated simulations showed that at 293 K, the critical times for the liquid finger-like structures were approximately 676 ps, 696 ps, and 696 ps, with an average of about 689 ps and a standard deviation of ±10 ps; at 368 K, the critical times were approximately 106 ps, 96 ps, and 96 ps, averaging around 99 ps with a standard deviation of ±5 ps. These results indicate that the key physical quantities reported in this study exhibit good statistical reproducibility, and the systematic shortening of the critical time for liquid finger-like structures with increasing temperature was consistently confirmed across the repeated simulations.

The affinity of CSH and SiO_2_ substrates for water differs, with the CSH side exhibiting stronger interaction than the SiO_2_ side. This leads to an asymmetric migration pattern of the solution in the early stage, characterized by higher fluid levels on the CSH side and lower ones on the SiO_2_ side. As the height difference between the two sides increases, the Laplace pressure difference across the gas–liquid interface at the center of the channel continuously accumulates. When this pressure exceeds the critical value supported by interfacial tension, the gas–liquid interface becomes unstable at locally weak points. Meanwhile, thermal fluctuations provide random triggers for local protrusions of the interface. Once the leading edge of the solution enters the van der Waals interaction range of silanol groups on the SiO_2_ surface, it drives the continuous extension of water chains toward the SiO_2_ side. The hydrogen-bonding network among water molecules enables the liquid finger to advance as a continuous chain. Increased temperature enhances the kinetic energy of water molecules, making it easier for thermal fluctuations to overcome interfacial tension constraints, thereby shortening the critical time required for liquid finger formation. This phenomenon indicates that groundwater transport through nanoscale fractures at the grout–rock interface does not proceed uniformly but instead achieves rapid breakthrough via preferential invasion of localized liquid fingers.

**Figure 6:**
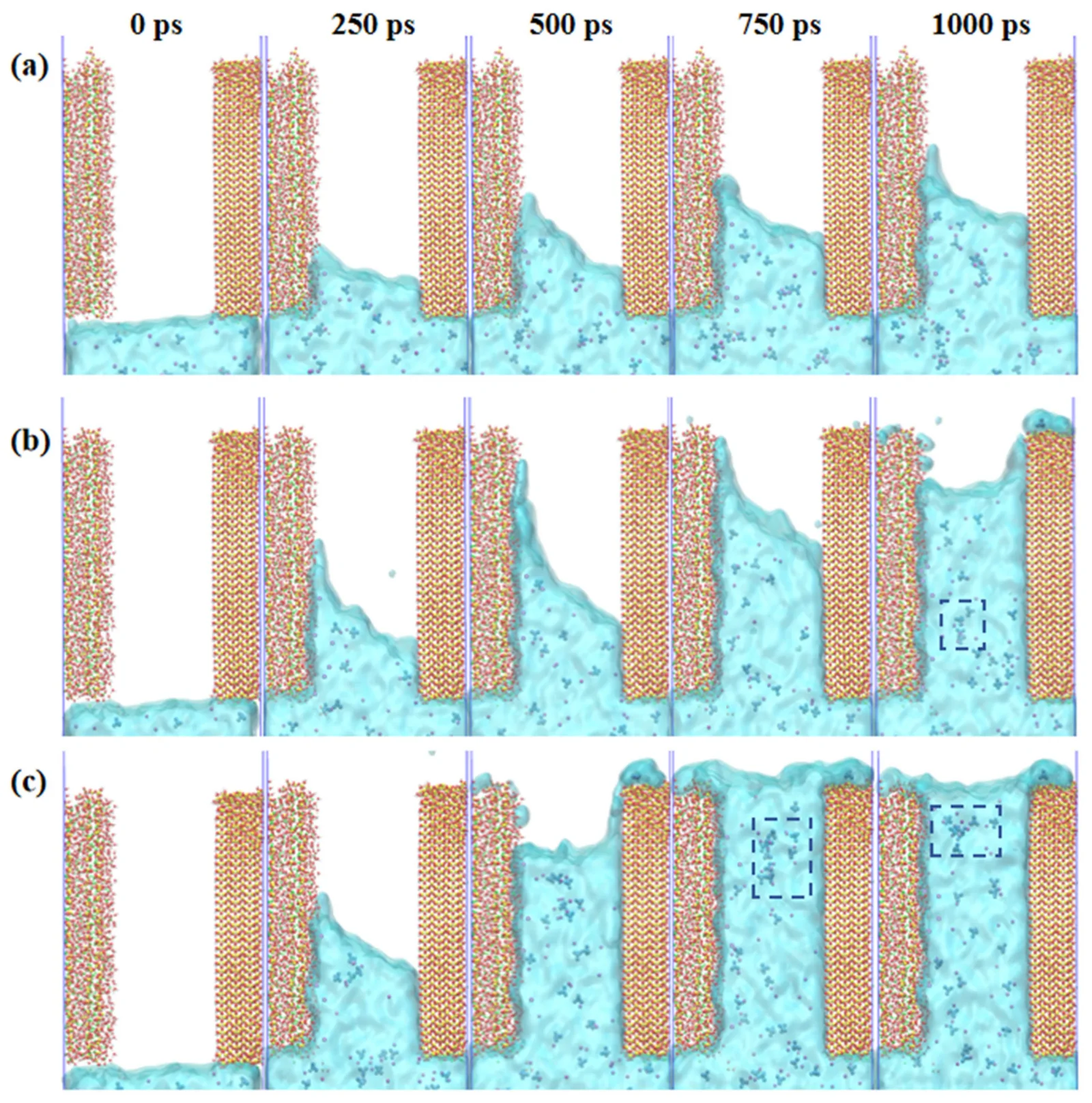
Snapshots of capillary flow of Na_2_SO_4_ solution (**a**) 293 K; (**b**) 323 K; (**c**) 353 K.

The migration process of Na_2_SO_4_ solution in CSH/SiO_2_ nanochannels is shown in Figure 6. Compared to pure water, the liquid front advancement speed in Na_2_SO_4_ solution is significantly reduced. At a temperature of 293 K, the aqueous solution has nearly filled the entire channel space by 1000 ps, while the Na_2_SO_4_ solution occupies only half of the channel volume. As water molecules diffuse into the channel, Na^+^ and SO_4_^2−^ ions migrate along with them, but their rising height consistently lags behind that of water molecules. Snapshots reveal that Na^+^ and SO_4_^2−^ ions tend to accumulate more on the CSH side. Under high-temperature conditions, distinct ion aggregation becomes evident, particularly at 368 K.

**Figure 7:**
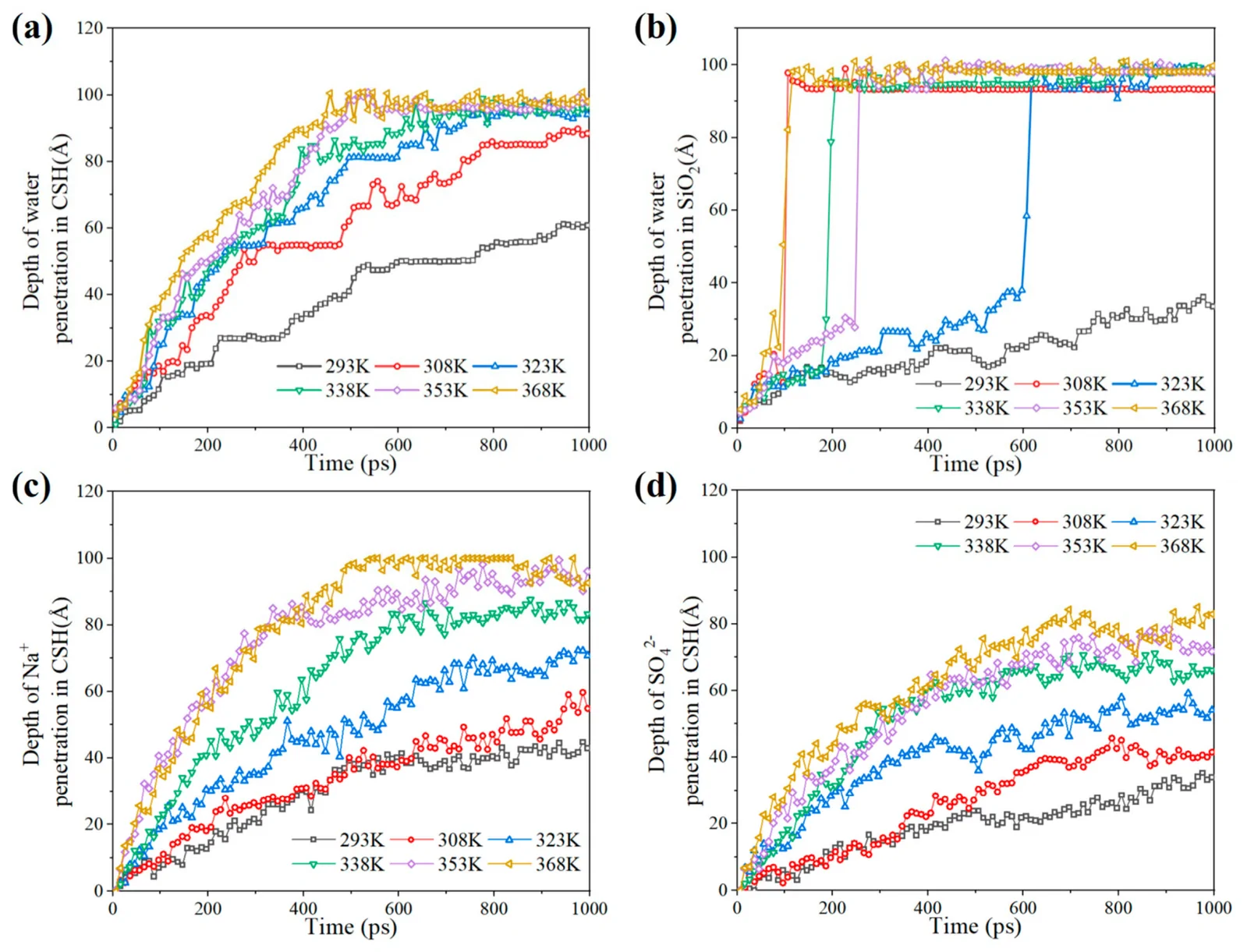
Penetration depths of water, Na^+^, and SO_4_^2−^ along the CSH and SiO_2_ surfaces at various temperatures. (**a**) water along the CSH surface, (**b**) water along the SiO_2_ surface, (**c**) Na^+^ along the CSH surface, and (**d**) SO_4_^2−^ along the CSH surface.

The penetration depth of aqueous solutions on the CSH and SiO_2_ sides as a function of simulation time is shown in Figure 7, with the invasion depth calculated based on the maximum migration distance of water molecules on each wall. In the CSH/SiO_2_ nanochannel, the permeation rates of various components follow the order: H_2_O > Na^+^ > SO_4_^2−^, which holds true for both walls, indicating that the selective ion blocking by the nanochannel is universal. This result is consistent with experimental findings reported in the literature [25]. Previous studies have suggested that this phenomenon is related to electrostatic interactions between ions and the channels of the CSH gel, leading to ion adsorption on the CSH channels and subsequent channel blockage [26].

As temperature increases, the migration of water molecules in the Na_2_SO_4_ system also accelerates. On the CSH side, the penetration depth reaches approximately 60 Å within about 1000 ps at 293 K, whereas it stabilizes near 95 Å after only around 500 ps at 368 K, as shown in Figure 7a. The penetration depths of Na^+^ and SO_4_^2−^ ions under different temperatures on the CSH side are illustrated in Figure 7c,d. At t = 1000 ps, the penetration depths of Na^+^ and SO_4_^2−^ ions in the 368 K environment reach 100 Å and 80 Å, respectively, significantly greater than the 40 Å and 30 Å observed at 293 K. Transport rates were calculated by dividing the distance increment by the time increment. For Na^+^ ions, the maximum and average transport rates at 368 K were 0.220 Å/ps and 0.154 Å/ps, respectively, compared to a maximum rate of 0.077 Å/ps and an average rate of 0.045 Å/ps at 293 K. Compared to lower temperatures, ions exhibit significantly faster average transport rates at higher temperatures, with an increase of 242.2% in average velocity; the same trend is observed for SO_4_^2−^ ions. Elevated temperatures reduce the energy barrier for ion migration, accelerating the deep penetration of aggressive media into the paste-rock interface. Meanwhile, the transport rate of cations (Na^+^) consistently exceeds that of anions (SO_4_^2−^), indicating preferential hindrance of ion migration at the interface.

**Figure 8:**
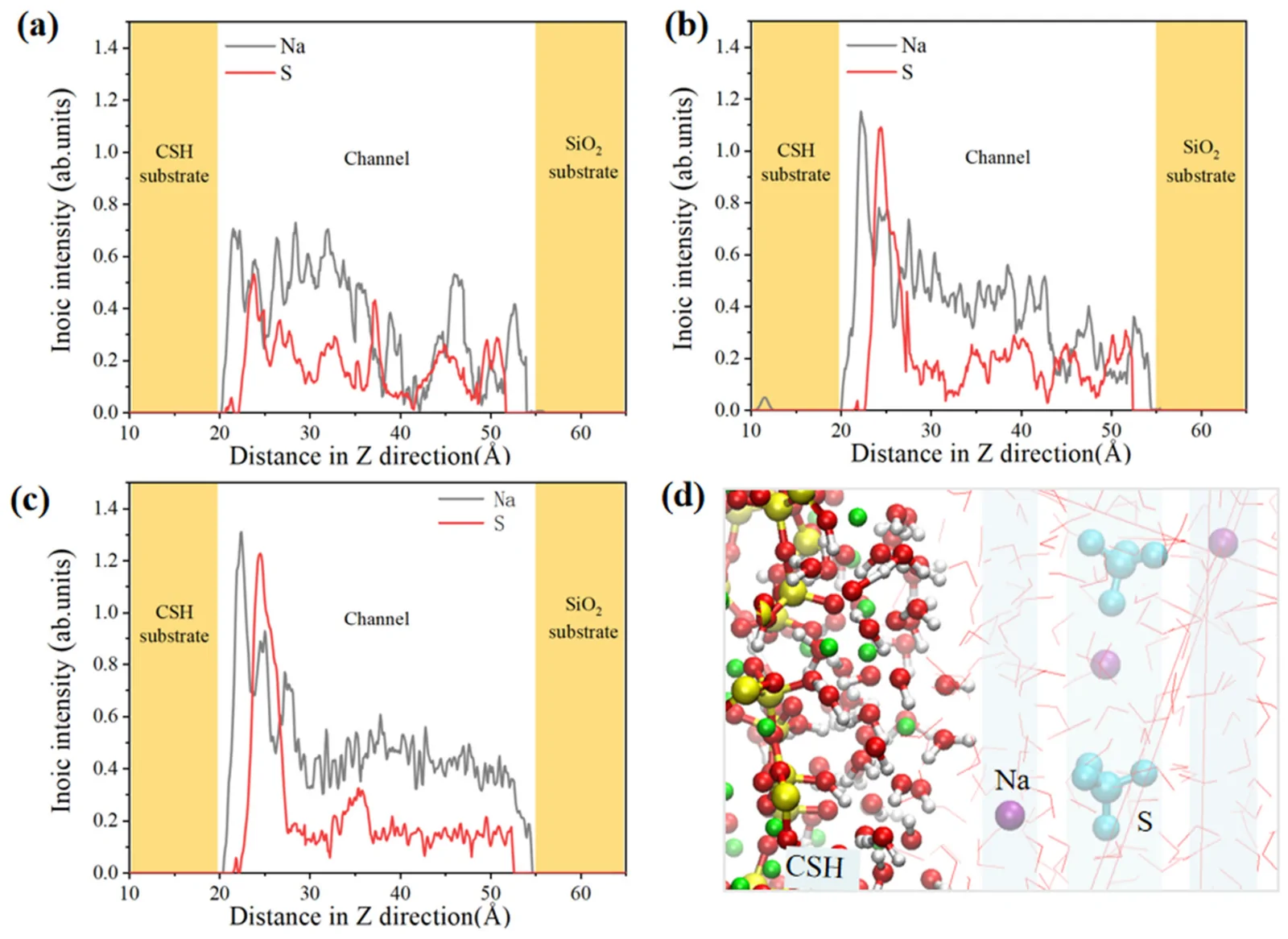
Density distributions of Na along the Z direction at (**a**) 293 K, (**b**) 323 K, and (**c**) 353 K, with (**d**) snapshot of ionic distribution near the CSH surface.

The density distribution profiles of Na^+^ and SO_4_^2−^ along the z-direction at different temperatures are shown in Figure 8. Both ions are primarily concentrated in the nanochannel region, forming distinct density peaks near the CSH and SiO_2_ surfaces, indicating ion enrichment at the solid–liquid interface. At all tested temperatures, the Na^+^ density peak on the CSH side is higher than that on the SiO_2_ side. This is because the negative charge and available interaction sites on the SiO_2_ surface are relatively weaker, resulting in slightly lower ion enrichment compared to the CSH side.

Under low-temperature conditions, both Na^+^ and SO_4_^2−^ exhibit high peaks on either side, but the peak intensity of Na^+^ is higher than that of SO_4_^2−^. Moreover, the peak positions of SO_4_^2−^ are further away from the density peaks of the two substrates compared to those of Na^+^, as shown in Figure 8a. According to the ion snapshots presented in Figure 8d, the peaks of Na^+^ and SO_4_^2−^ are orderly located near the two substrates, with sodium ions and sulfate ions accumulating layer by layer on the substrate surface. This occurs because the negatively charged CSH surface attracts Na^+^ via electrostatic forces, bringing them close to the interface while repelling sulfate ions. Although SO_4_^2−^ is repelled, it can still indirectly accumulate near the interface by forming ion pairs with Na^+^. Consequently, anions and cations are arranged in an ordered layered structure within the channel. As temperature increases, as shown in Figure 8b,c, the density peaks of both ions at the interface become further enhanced, while the ion concentration in the central region of the channel decreases accordingly. Some ions that were previously freely diffusing gradually transition into adsorbed states at the interface, forming localized ion clusters.

The temperature-induced ion enrichment phenomenon is related to changes in the interactions between ions and water molecules, as well as between ions and solid surfaces. Heating alters the hydration structure around Na^+^ and SO_4_^2−^, making some ions more accessible to the solid surface. Meanwhile, the association between Na^+^ and SO_4_^2−^ ions, along with their interactions with reactive oxygen sites on the substrate surface, collectively promote the formation of ion clusters. The underlying mechanisms will be further analyzed using radial distribution functions.

### 3.2. Local Structure of Different Solutions in CSH/SiO_2_ Channels

To quantitatively clarify the difference in water molecule adsorption capacity between the two surfaces, this study calculated the interaction energy between the CSH/H_2_O and SiO_2_/H_2_O systems using Equation (1).

(1) $$E_{1/2}=\frac{E_{\mathrm{total}}-(E_{1}+E_{2})}{A_{1/2}},$$

where *A*_1/2_ denotes the interfacial contact area between components 1 and 2; *E*_1/2_ is the interfacial interaction energy density (i.e., energy per unit area) between them; *E*_total_ refers to the total potential energy of the combined system comprising both components; and *E*_1_ and *E*_2_ represent the isolated energies of component 1 and component 2, respectively.

**Figure 9:**
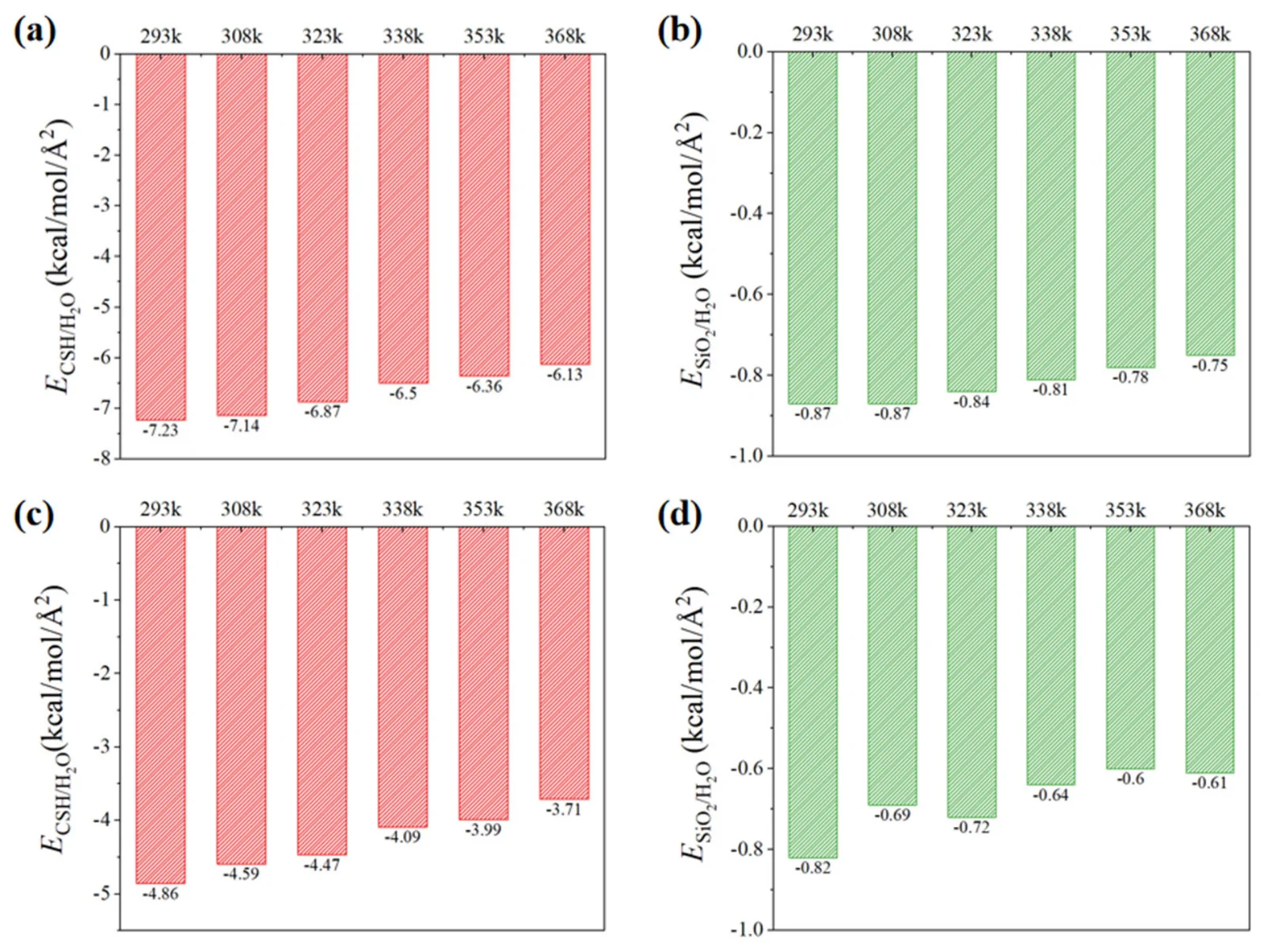
The interaction energy of (**a**) CSH/H_2_O, (**b**) SiO_2_/H_2_O in water, and (**c**) CSH/H_2_O, (**d**) SiO_2_/H_2_O in Na_2_SO_4_ solution.

As shown in Figure 9a,b, *E*_CSH/H2O_ = −7.23 is approximately eight times greater than *E*_SiO2/H2O_ = −0.87, indicating a significant difference that confirms the stronger attraction of CSH to water molecules compared to SiO_2_. The surface of CSH contains numerous exposed calcium sites and abundant hydroxyl groups, enabling the formation of a strong hydrogen-bonding network with water molecules. This high interfacial binding energy provides the primary driving force for wetting and migration of water molecules on the CSH surface. Through cooperative transmission via the hydrogen-bonding network, water molecules form continuous and stable migration pathways along the CSH surface, facilitating preferential upward movement. In contrast, although the SiO_2_ surface has been hydroxylated and possesses certain polar sites, the number of active sites is limited and the interaction strength with water molecules is relatively weak, preventing the formation of a hydrogen-bonding network density comparable to that of CSH. Due to insufficient surface adsorption forces, water molecules struggle to establish continuous and stable migration channels on the SiO_2_ side, resulting in significantly lower migration height and diffusion rate compared to the CSH side, and exhibiting overall more dispersed and slower migration behavior. These energetic differences provide evidence for the preferential ascent of aqueous solutions on the CSH side and the delayed response on the SiO_2_ side.

After introducing Na_2_SO_4_, the absolute values of water-substrate interaction energies on both surfaces decreased, indicating that the presence of ions to some extent weakens the attraction between water molecules and the solid surface, as shown in Figure 9c,d. This phenomenon arises from a combination of competitive ion adsorption and electrostatic shielding effects. Upon entering the channels, Na^+^ and SO_4_^2−^ not only hydrate with water molecules but also tend to accumulate near the solid surface, occupying space originally available for water molecules to interact with the substrate, thereby reducing the adsorption capacity of interfacial water molecules. Meanwhile, this adsorbed layer reduces the polarization effect of surface charges on water molecules through electrostatic shielding, thus lowering the binding energy of interfacial water. Furthermore, the interaction energy on the CSH side remains higher than that on the SiO_2_ side, indicating that its adsorption advantage over water molecules is not eliminated by the presence of ions.

The radial distribution function (RDF) is a core analytical method in molecular dynamics simulations for characterizing the local atomic structure of a system. It enables quantitative assessment of interfacial bonding strength and stability by revealing bond lengths, coordination numbers, and distribution ranges of ionic and hydrogen bonds at interfaces. A higher RDF peak indicates stronger attractive interactions between ion pairs. The position of the peak directly correlates with bond length-the earlier the peak appears, the shorter the bond length, and the more stable the structure [46].

**Figure 10:**
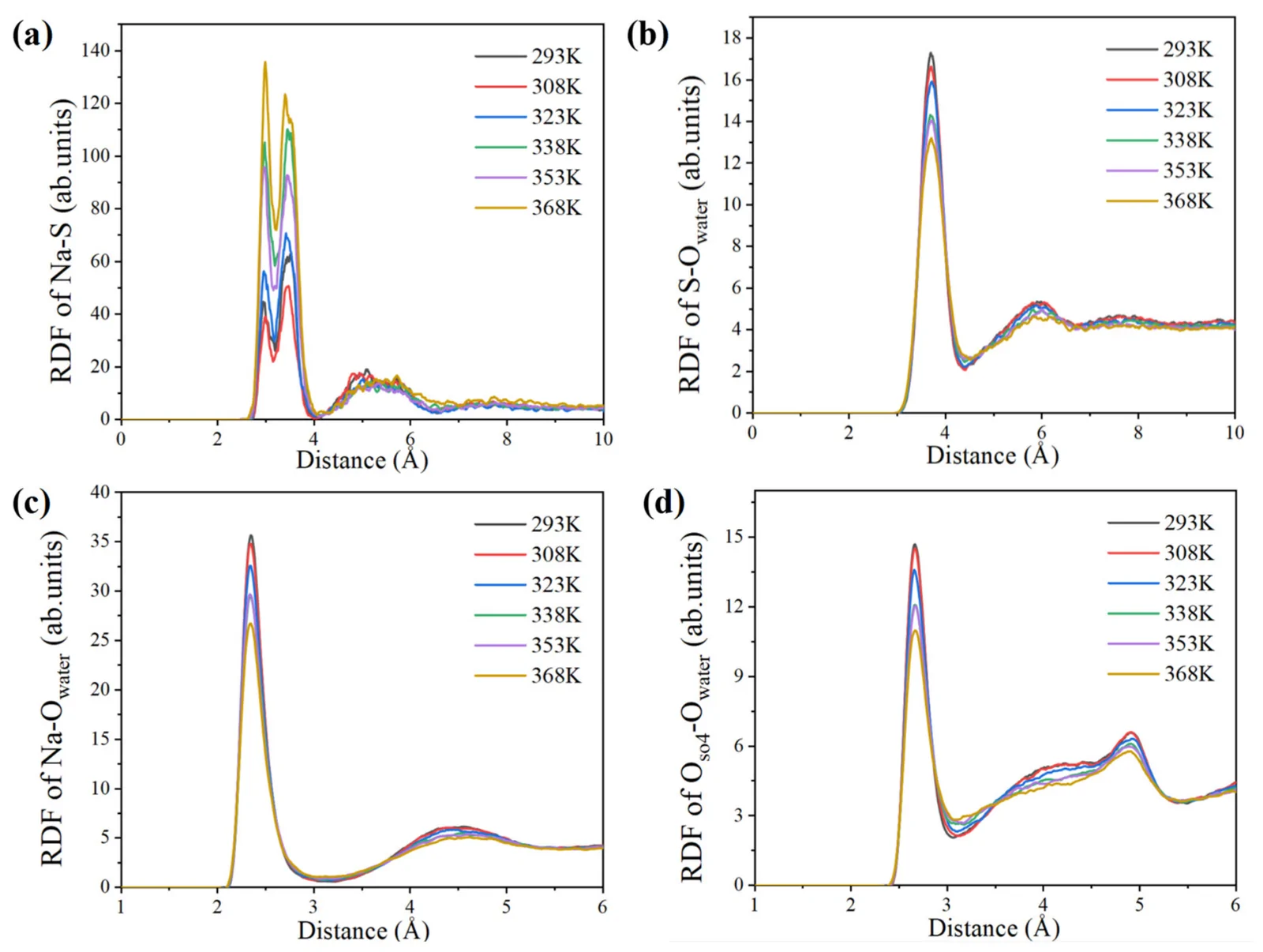
The RDF curves of (**a**) Na-S, (**b**) S-O_water_, (**c**) Na-O_water_, and (**d**) O_SO4_-O_water_.

The microevolution of ion hydration structures and their response to temperature are shown in Figure 10 and Table 1. At 293 K, the first-shell coordination number and first hydration radius for Na^+^ are 4.35 and 240 pm, respectively, showing high consistency with experimental diffraction results that report hydration numbers of 4–8 and first-shell radii of 240–250 pm [47]. The first-shell hydration coordination number for S and Ow is 11.43, which falls within the range of 7–12 water molecules obtained from X-ray diffraction, thereby supporting the accuracy of the molecular dynamics simulations used in this study [48].

Figure 10a,b show the radial distribution functions (RDFs) of Na-Owater and S-Owater, respectively. The first characteristic peaks for Na-Owater and S-Owater appear at 2.3 Å and 3.7 Å, while the second peaks occur at 4.5 Å and 5.7 Å. The peak intensities of both RDF curves decrease with increasing temperature. Short-range and medium-range spatial correlations gradually weaken as temperature rises, indicating a reduction in the number of water molecules in the first and second hydration shells. As shown in Table 1, when the temperature increases from 293 K to 353 K, the hydration numbers of the first hydration shell around sodium and sulfate ions decrease by 0.63 and 1.28, respectively. The rise in temperature intensifies the disruption of the hydration hydrogen-bond network; water molecules gain higher thermal kinetic energy, leading to increased mobility and causing some water molecules to detach from the ion hydration shells. The area of the first peak in the sulfate oxygen-water oxygen RDF curve shown in Figure 10d decreases with rising temperature, confirming the disruptive effect of thermal fluctuations on the hydration hydrogen-bond network, consistent with previous studies [49]. As the hydration shell weakens, its confining effect on ions diminishes accordingly, increasing the effective collision frequency between cations and anions, thereby favoring the formation of ion pairs.

It should be noted that all RDF values were calculated using the standard LAMMPS implementation and normalized consistently based on the average number density of each species within the simulation box. Therefore, the relatively high Na-S RDF peak should be attributed to the strong spatial correlation and local enrichment of Na^+^ and SO_4_^2−^ under nanochannel confinement, rather than to errors introduced during the normalization process.

In Figure 10c, a distinct first coordination peak between Na^+^ and SO_4_^2−^ is evident, confirming the existence of ion association between them in solution. The peak intensity gradually increases with rising temperature, with the coordination numbers for Na-S and S-Na increasing to 1.19 and 2.36, respectively. As temperature rises, the hydration structure of ions weakens progressively, granting Na^+^ and SO_4_^2−^ greater mobility and facilitating more frequent collisions that lead to ion association. Enhanced spatial correlation between Na and S further promotes the formation of local ion clusters, causing more ions to accumulate near the solid–liquid interface. This result corroborates the interfacial enrichment observed in the previous ion density distribution, indicating that while elevated temperature enhances ionic mobility, it also promotes localized ion aggregation.

**Figure 11:**
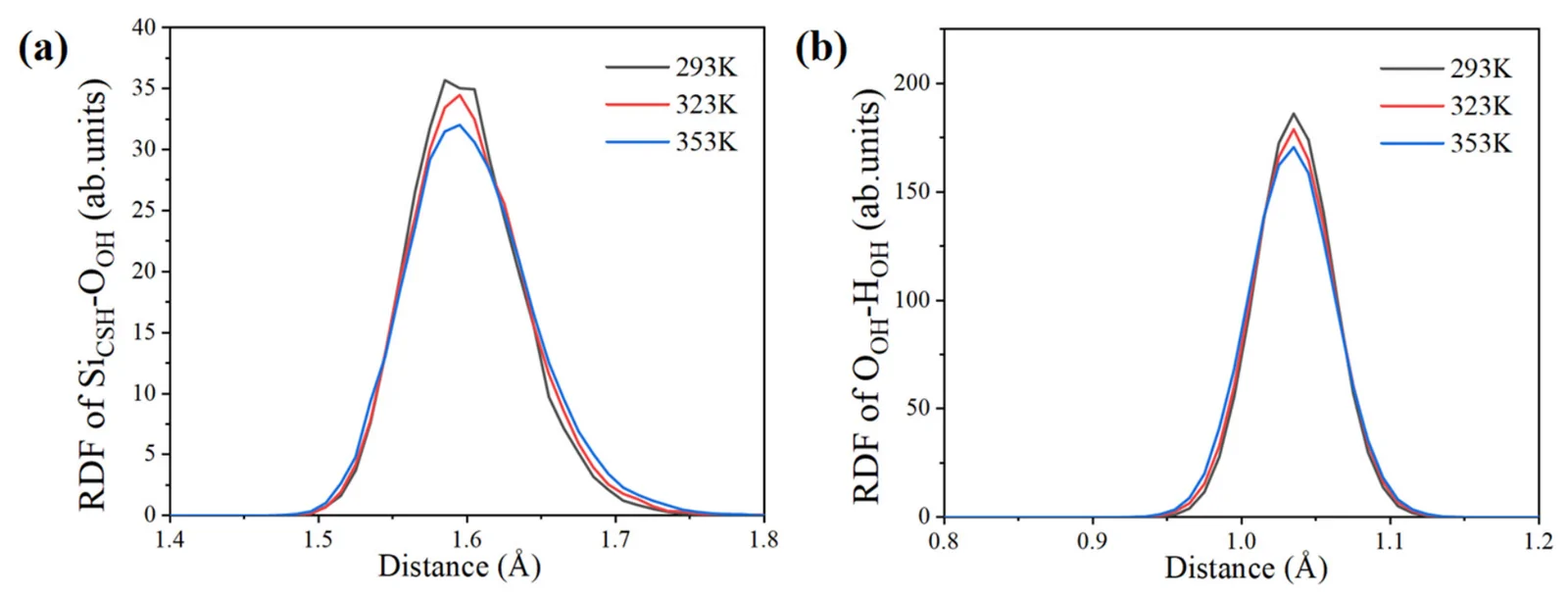
The RDF curves of (**a**) Si_CSH_-O_OH_, and (**b**) O_OH_-H_OH_.

Temperature increase also induces changes in the CSH substrate structure. Figure 11 shows the radial distribution functions (RDFs) of Si-O and Os-Hs bonds. By analyzing the positions of the first coordination peaks in the corresponding RDFs, bond lengths can be determined. The bond lengths remain stable between 293 K and 368 K, maintaining at approximately 1.595 Å and 1.035 Å, respectively. However, the shoulder regions of the RDFs for both types of bonds exhibit a gradual broadening with increasing temperature. Specifically, the shoulder region of the Si-O_OH_ bond widens from 0.25 Å to 0.29 Å, while that of the O_OH_-H_OH_ bond expands from 0.17 Å to 0.21 Å. This indicates that both the siloxane network and surface hydroxyl structures are subject to stronger thermal disturbances, leading to increased bond vibrational amplitudes and reduced local ordering and stability.

**Figure 12:**
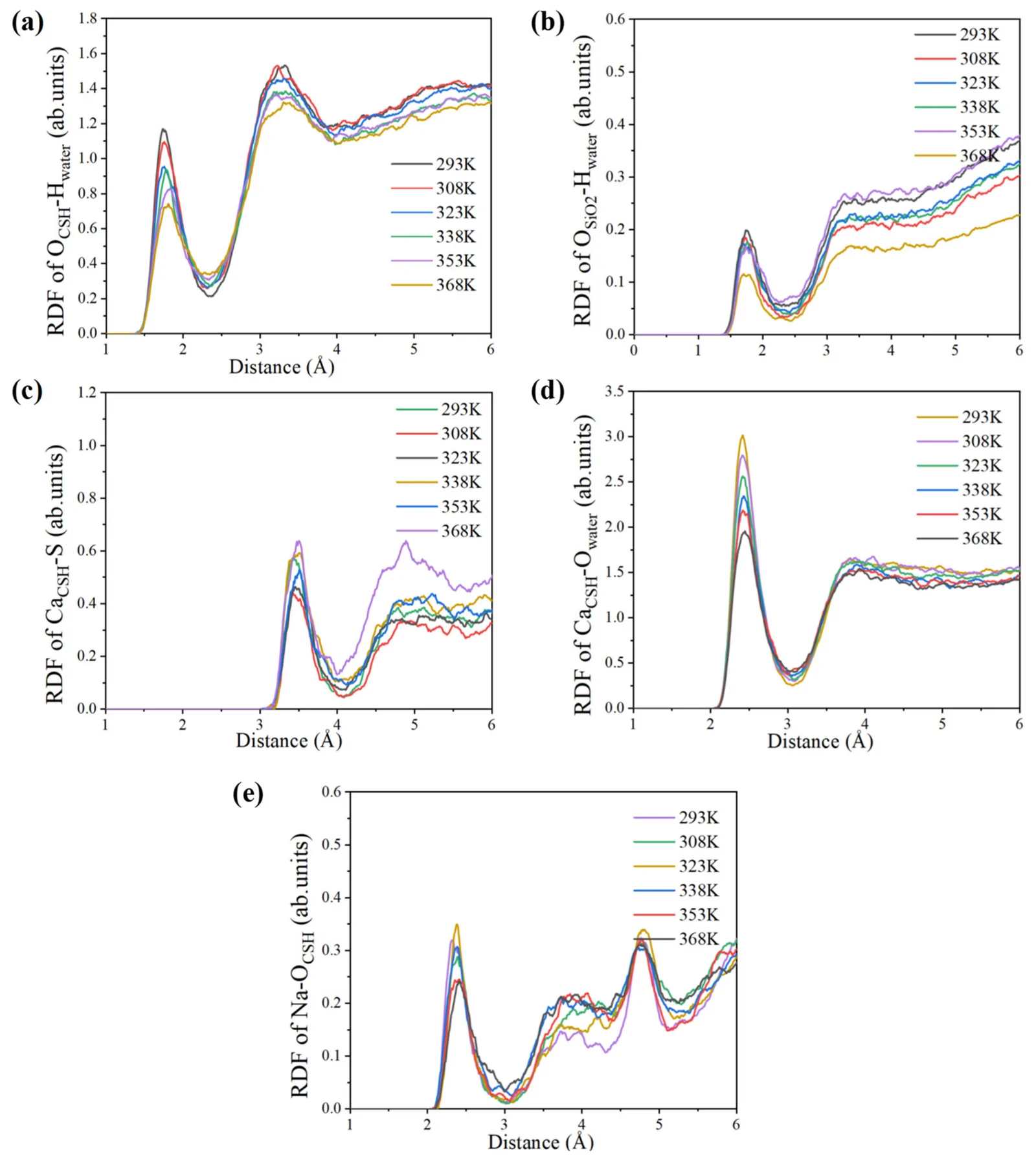
The RDF curves of (**a**) O_CSH_-H_water_, (**b**) O_SiO2_-H_water_, (**c**) Ca_CSH_-S, (**d**) Ca_CSH_-O_water_, and (**e**) Na-O_CSH_.

The structural changes in the CSH framework, along with the stretching vibrations of Si-O and O-H bonds, enhance the chemical reactivity of certain oxygen sites on the CSH surface. As a result, aqueous solutions and Na^+^ ions are more readily electrostatically attracted to the CSH substrate. As shown in Figure 12a,b, both the O_CSH_-H_water_ and O_SiO2_-H_water_ RDFs exhibit prominent first peaks at approximately 1.7 Å, indicating that water molecules can adsorb onto both substrates via hydrogen bonding. The peak intensity of O_CSH_-H_water_ is significantly higher than that of O_SiO2_-H_water_, demonstrating stronger water adsorption on the CSH surface compared to SiO_2_, consistent with the previously observed interaction energy results. With increasing temperature, the RDF peak intensities for both interfaces gradually decrease, as thermal fluctuations disrupt the local ordered structure between water molecules and the solid surface, weakening interfacial hydrogen bonding. In contrast to water molecules, however, the RDF values for ions at the interface increase upon heating. As illustrated in Figure 12e, the spatial correlations between Na^+^ and OCSH at distances of approximately 2–3 Å and 4–6 Å become stronger with rising temperature, indicating enhanced interactions and increased accumulation of sodium ions within the channels. This also explains why the CSH surface becomes more favorable for Na^+^ adsorption at elevated temperatures, aligning with the interfacial enrichment phenomenon observed in the ion density distributions discussed earlier [50].

In addition to the interaction between water molecules and oxygen sites, calcium in the CSH structure also participates in the interfacial adsorption process. As shown in Figure 12c, the Ca_CSH_-S RDF curve exhibits a characteristic peak near 3.4 Å, indicating that SO_4_^2−^ can adsorb onto the solid surface by interacting with calcium sites in CSH. The peak intensity increases with rising temperature, suggesting that higher temperatures promote the migration and enrichment of SO_4_^2−^ toward the CSH interface. The Ca_CSH_-O_water_ RDF plot reveals strong association between calcium sites and water molecules, demonstrating that calcium sites on the CSH surface simultaneously participate in both water adsorption and ion immobilization, as illustrated in Figure 12d.

Rising temperature weakens the hydrogen bonding between water and the CSH surface, while simultaneously enhancing the interactions between Na^+^, SO_4_^2−^ ions and the CSH surface, promoting ion enrichment at the interface. These adsorbed ions not only occupy space previously available for water molecule migration but also form local aggregated structures through ionic association, further restricting continuous pathways for water movement, ultimately leading to a reduced water transport rate in the Na_2_SO_4_ system.

Radial distribution functions (RDF) and interface interaction analysis mainly reflect the local structural characteristics of water molecules and ions near solid–liquid interfaces. However, fluid transport within nanochannels is essentially a dynamic evolution process, making it difficult to fully describe their migration behavior using only static structural parameters. To address this, this study further employs mean square displacement (MSD) to conduct kinetic analysis of the diffusion processes of water molecules and ions.

### 3.3. Water and Ion Transport Kinetics at Different Temperatures

MSD describes the average displacement change in particles relative to their initial positions over a given time interval and can be used to evaluate the migration ability of different components within a system. During the diffusion stage, the growth rate of the MSD curve is closely related to the particle’s diffusion coefficient, the steeper the slope, the higher the particle’s diffusivity and migration rate. By comparing the MSD evolution patterns of water molecules and ions under different temperatures and solution conditions, the effects of temperature and ion-interface interactions on transport behavior in nanochannels can be further revealed.

(2) $$M(t)=\langle {\left|r_{i}(t)-r_{i}(0)\right|}^{2}\rangle ,$$

Here, *r_i_*(0) denotes the initial position of a particle, and *r_i_*(*t*) represents the position of the same particle at time *t*; angle brackets indicate the ensemble average over all particles of the same type.

It should be noted that the MSD calculated during capillary imbibition may simultaneously include contributions from molecular diffusion and capillary-driven convection, due to the non-equilibrium nature of the transport process. Therefore, MSD is used here primarily as a comparative indicator of molecular mobility at different temperatures, rather than a direct measurement of self-diffusion.

**Figure 13:**
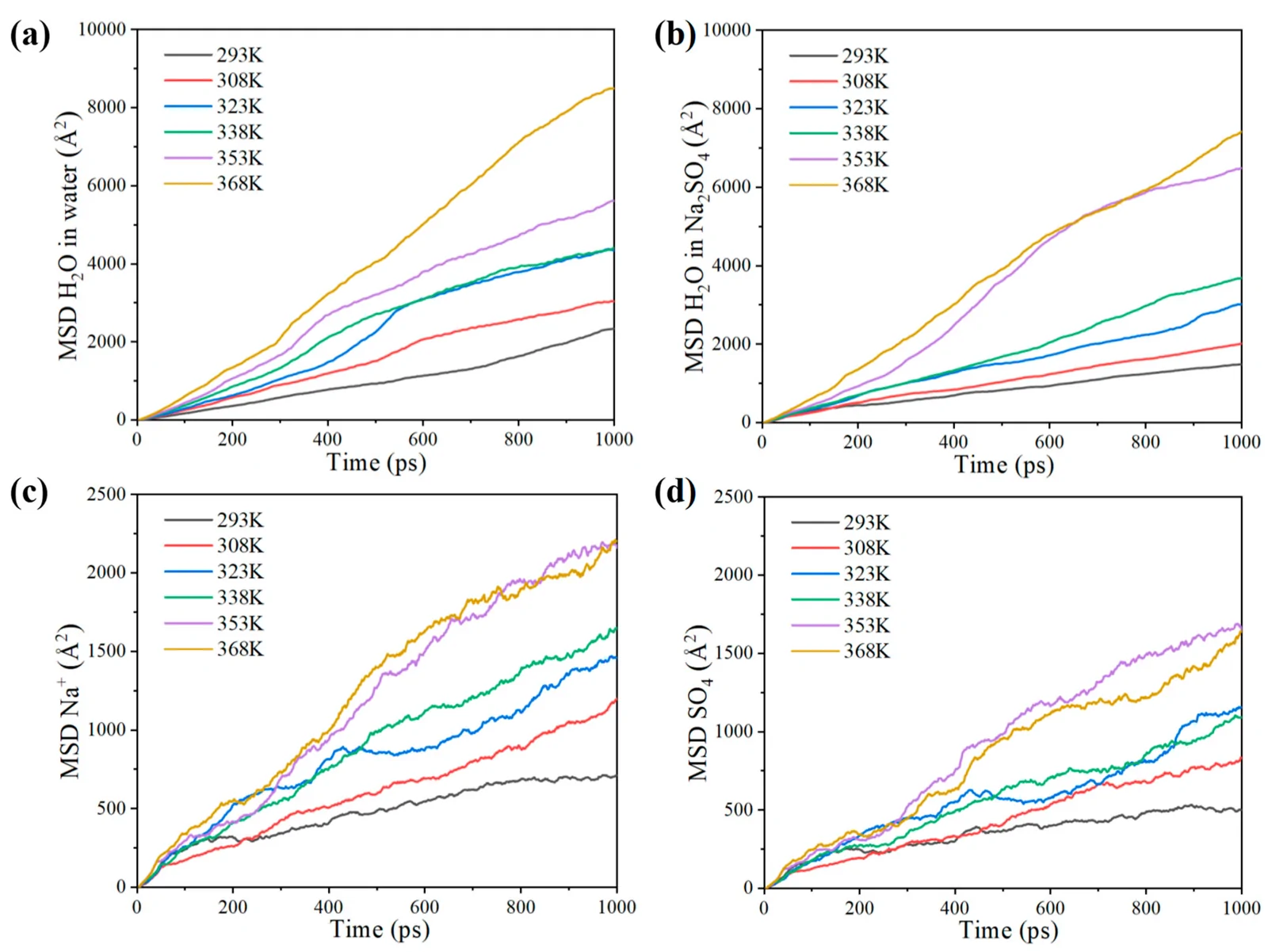
MSD of (**a**) H_2_O in water, (**b**) H_2_O in Na_2_SO_4_ solution, (**c**) Na^+^, and (**d**) SO_4_^2−^.

The mean square displacement (MSD) curves of water molecules and ions are shown in Figure 13. In the pure water system, as illustrated in Figure 13a, the MSD of water molecules increases continuously with time, and the slope gradually becomes steeper with rising temperature. At 293 K, the MSD growth is slowest, reaching only 2100 Å^2^ at 1000 ps, whereas at 368 K, the MSD reaches approximately 8500 Å^2^, significantly higher than that at lower temperatures. Heating enhances the thermal motion of water molecules. The relatively low molecular mass and high rotational degrees of freedom of water molecules, combined with the rapid dynamic equilibrium of the hydrogen-bonding network, enable them to maintain high kinetic activity within the channels. However, excessively high diffusion rates inevitably lead to unstable bonding and excessive mobility, resulting in a lower probability of forming stable hydrogen bonds with the surrounding matrix at elevated temperatures compared to lower temperatures.

In the Na_2_SO_4_ solution environment, as shown in Figure 13b, the mean square displacement (MSD) of water molecules at all temperatures is lower than that in pure water, reaching approximately 1500 Å^2^ at 293 K and about 7400 Å^2^ at 368 K. Notably, the difference in MSD values between the two systems decreases from 30% at low temperatures to 13% at higher temperatures. Water molecules in the salt solution actually exhibit greater sensitivity to temperature changes compared to pure water. At elevated temperatures, the stability of ion hydration shells weakens, as indicated by the reduced peak intensities in the RDF plots of Figure 7b,c. A large number of water molecules originally confined within the ion coordination layers are released as free water, thereby increasing the population of mobile water molecules.

There are significant differences in the diffusion rates of various particles, with migration ability decreasing in the order: H_2_O > Na^+^ > SO_4_^2−^. The MSD values of both Na^+^ and SO_4_^2−^ increase with rising temperature, but their growth rates are much smaller than those of water molecules, as shown in Figure 13c,d. At 293 K, the MSD values of Na^+^ and SO_4_^2−^ are 700 Å^2^ and 500 Å^2^, respectively, increasing to 2200 Å^2^ and 1600 Å^2^ at 368 K. In contrast, the MSD of water molecules increases from 2300 Å^2^ to 8500 Å^2^ over the same temperature range, representing a 3.7-fold increase, significantly greater than the 3.1-fold increase for Na^+^ and the 3.2-fold increase for SO_4_^2−^. The response of ion diffusion to temperature is relatively weak, consistent with previous findings on ion density distribution and RDF results. Although heating enhances ion mobility, it also weakens their hydration structures, promoting ion pair formation and interfacial enrichment.

It should be noted that the current CSH/SiO_2_ nanochannel model employs an idealized, smooth geometry with a constant width of 3.5 nm, neglecting phenomena such as surface roughness, channel size heterogeneity, and mineral composition variations present at actual cement-rock interfaces. This simplification is necessary to isolate the fundamental mechanisms of fluid transport at the atomic scale, and such approaches have been widely used in nanoscale transport studies [26]. Therefore, the transport behaviors and characteristic times obtained in this study should be interpreted as mechanistic insights rather than direct quantitative predictions for real-scale behavior. To bridge the gap between these atomic-level findings and macroscopic engineering performance, future work must incorporate multiscale modeling that accounts for more realistic pore structures and interfacial characteristics.

The atomistic data generated in this study provides a foundation for future development of machine learning surrogate models or Bayesian optimization frameworks. Existing data, including penetration depth, molecular mobility, ion hydration, and interfacial interaction energies, can serve as training data for such models to efficiently predict transport properties under a broader range of conditions, including temperature, ion concentration, solution composition, pore geometry, and surface chemistry. This approach holds promise for partially alleviating the computational cost limitations of molecular dynamics simulations and offers a potential pathway to bridge idealized nanochannel models with the complex conditions encountered in real engineering applications.

## 4. Conclusions

This study employs molecular dynamics simulations to investigate the migration behavior of water molecules and ions in CSH/SiO_2_ nanochannels under different temperatures and Na_2_SO_4_ environments. By combining analyses of penetration depth, interfacial interactions, radial distribution functions, and mean square displacement, the mechanisms by which temperature and ion effects influence fluid transport are revealed. The main conclusions are as follows:

(1) Water migration within CSH/SiO_2_ nanochannels exhibits significant wall-dependent differences, with water molecules preferentially diffusing along the CSH side. At 293 K, the adsorption energy *E*_CSH/H2O_ = −7.23 is approximately eight times greater than *E*_SiO2/H2O_ = −0.87. This strong interfacial adsorption arises primarily from Ca sites and hydroxyl groups on the CSH surface, which enhance the attraction toward water molecules and ions.
(2) Unlike the continuous migration pattern observed on the CSH side, water transport on the SiO_2_ side exhibits distinct stage-like characteristics. Water preferentially migrates along the CSH side; as the penetration depth difference between the two sides increases, local water molecules extend toward the SiO_2_ side, forming liquid finger-like protrusions that rapidly fill cavities and establish continuous pathways in a short time. Heating significantly advances the critical time for liquid finger formation, shifting it from 700 ps at 293 K to just 100 ps at 368 K.
(3) Elevated temperature generally promotes the migration of water molecules. In pure water, the mean square displacement (MSD) of water molecules at 368 K is approximately 8500 Å^2^, compared to only about 2300 Å^2^ at 293 K, indicating that heating enhances the thermal motion capability of water molecules. In contrast, water transport is significantly suppressed in the presence of Na_2_SO_4_, with MSD decreasing to around 7400 Å^2^ at 368 K. Within the channel, the migration rates of components follow the order H_2_O > Na^+^ > SO_4_^2−^, with water molecules consistently migrating faster than ions. The increase in ion diffusion due to temperature rise is smaller than that of water molecules.
(4) The introduction of Na_2_SO_4_ ions alters the transport mechanism within the nanochannel. Both Na^+^ and SO_4_^2−^ exhibit distinct density peaks near the CSH and SiO_2_ walls, with higher ion enrichment observed on the CSH side compared to the SiO_2_ side. At the interface, the Na^+^ peak is closer to the matrix than the SO_4_^2−^ peak, indicating a distribution pattern characterized by cation inner-layer adsorption and anion outer-layer coordination. Upon heating, the ion density peaks at the interface further increase, while the hydration structure of the ions weakens, enhancing the association between Na^+^ and SO_4_^2−^. This promotes the accumulation of certain ions toward the interface and leads to the formation of localized ionic structures.

In summary, this study investigated the dynamic transport behavior of pure water and Na_2_SO_4_ solution in CSH/SiO_2_ nanochannels under different temperature conditions using molecular dynamics simulations. Although the simulation results are limited to nanoscale spatial and nanosecond temporal dimensions, the revealed fundamental mechanisms hold engineering relevance. Specifically, the simulations show that Na^+^ and SO_4_^2−^ ions adsorb and accumulate at the CSH surface, with this tendency significantly enhanced by increasing temperature. The continuous penetration of SO_4_^2−^ is atomistically characterized by interactions with Ca sites on the CSH surface, consistent with experimental observations of sulfate attack leading to decalcification, structural degradation, and strength deterioration of CSH gel. On a macroscopic scale, the cumulative effect of such erosion often manifests as the precipitation of gypsum and other corrosion products on the surface of cement-based materials, aligning with typical deterioration patterns observed in grouting projects under sulfate-rich environments. Therefore, the nano-scale insights into ionic transport and interfacial aggregation provided by this study offer an atomic-level physical basis for understanding the degradation mechanisms of durability at the grout–rock interface under high-temperature, water-rich conditions.

## Figures and Tables

**Table 1:** Coordination numbers of Na and S at different temperatures.

	Na				S			
	Na-O_water_	Na-S	Na-O_CSH_	Total	S-O_water_	S-Na	S-Ca	Total
293 K	4.35	0.83	0.19	5.37	11.43	1.69	0.08	13.20
323 K	3.81	1.07	0.25	5.13	10.82	2.21	0.15	13.18
353 K	3.72	1.19	0.27	5.18	10.15	2.36	0.17	13.08

## Data Availability

The original contributions presented in this study are included in the article. Further inquiries can be directed to the corresponding author.
